# Dietary Patterns and Long-Term Outcomes in Patients with NAFLD: A Prospective Analysis of 128,695 UK Biobank Participants

**DOI:** 10.3390/nu15020271

**Published:** 2023-01-05

**Authors:** Zhening Liu, Hangkai Huang, Jiarong Xie, Chengfu Xu

**Affiliations:** 1 Department of Gastroenterology, The First Affiliated Hospital, Zhejiang University School of Medicine, Hangzhou 310003, China; 2 Department of Gastroenterology, Ningbo First Hospital, Ningbo 315010, China; 3 Zhejiang Provincial Clinical Research Center for Digestive Diseases, Hangzhou 310003, China

**Keywords:** non-alcoholic fatty liver disease, end-stage liver disease, mortality, dietary pattern

## Abstract

Large longitudinal studies exploring the role of dietary patterns in the assessment of long-term outcomes of NAFLD are still lacking. We conducted a prospective analysis of 128,695 UK Biobank participants. Cox proportional hazards models were used to estimate the risk associated with two dietary patterns for long-term outcomes of NAFLD. During a median follow-up of 12.5 years, 1925 cases of end-stage liver disease (ESLD) and 12,466 deaths occurred in patients with NAFLD. Compared with patients in the lowest quintile, those in the highest quintile of the diet quality score was negatively associated with the risks of ESLD and all-cause mortality (HR_Q5vsQ1_: 0.76, 95% CI: 0.66–0.87, *p* < 0.001; HR_Q5vsQ1_: 0.84, 95% CI: 0.79–0.88, *p* < 0.001, respectively). NAFLD patients with high-quality carbohydrate patterns carried a 0.74-fold risk of ESLD and a 0.86-fold risk of all-cause mortality (HR_Q5vsQ1_: 0.74, 95% CI: 0.65–0.86, *p* < 0.001; HR_Q5vsQ1_: 0.86, 95% CI: 0.82–0.91, *p* < 0.001, respectively). For prudent dietary patterns rich in vegetables, fruits and fish, the adjusted HR _Q5vsQ1_ (95% CI) was 0.87 (0.76–0.99) and 0.94 (0.89–0.99) for ESLD and all-cause mortality of NAFLD patients. There was a U-shaped association between the meat-rich dietary pattern and all-cause mortality in patients with NAFLD. These findings suggest that a diet characterized by a high-quality, high intake of vegetables, fruits, fish and whole grains as well as an appropriate intake of meat, was associated with a lower risk of adverse outcomes of NAFLD.

## 1. Introduction

Nonalcoholic fatty liver disease (NAFLD) is a very prevalent but widely underappreciated liver disease that is closely related to other metabolic disorders and was therefore called metabolic dysfunction-associated fatty liver disease (MAFLD) [1]. The global prevalence of NAFLD was estimated to be 32.4% [2]. The dramatically increased disease burden of NAFLD has been propelled by the progressively severe health effects of obesity and type 2 diabetes [3]. NAFLD covers a range of liver conditions, from simple steatosis to steatohepatitis and fibrosis, the latter of which carries a higher risk of developing end-stage liver disease [4]. The incidence rate of hepatocellular carcinoma parallels the severity of NAFLD, rising from 0.15 to 14.46 and 19.13 per 1000 person-years for steatosis, fibrosis and cirrhosis, respectively [5]. Moreover, NAFLD patients have a significantly increased risk of overall mortality, and this risk changes in tandem with the histology stage of NAFLD [6]. For the majority of patients, NAFLD is a benign condition [7]. However, advanced liver disease is usually diagnosed late, and interventions at this stage are less effective than earlier treatments [8]. A critical challenge is to identity NAFLD patients at higher risk of progressive liver disease so that early interventions could be targeted to those most in need [9].

A lifestyle modification focused on diet and exercise is the first-line treatment for NAFLD [10]. Diet intervention has raised great interest around the world. Previous studies reported the significant relationship between several nutrients and NAFLD, such as red meat consumption [11] being positively associated with the risk of NAFLD while yogurt [12] and soy milk [13] being inversely associated with the risk of NAFLD. Nevertheless, one does not eat a single nutrient but a complex mixture of foods which interact with each other [14]. Exploring the separate effects of isolated nutrient components does not represent well the dietary habits in the real world. Dietary patterns, by contrast, take the contributions of various aspects of foods into account and therefore more closely reflect the habitual diet in real-life settings [15]. Dietary patterns are usually derived by two methods: a priori and a posteriori method [16]. The a priori approach is based on hypotheses according to dietary guidelines about whether foods are favorable or unfavorable. The a posteriori approach is an exploratory analysis that accounts for variation in the habitual intake in a specific population.

To date, only a few small cross-sectional studies were performed to investigate the relationship of dietary patterns with NAFLD risks [17]. Previous studies showed that a high diet quality, assessed by the alternate healthy eating index (AHEI), was inversely associated with hepatic steatosis [18]. In addition, a prudent dietary pattern was associated with an odds ratio of 0.78 for NAFLD, whereas the Western dietary pattern was associated with a 1.56-fold increased risk of NAFLD [17]. Large longitudinal studies exploring the role of dietary patterns in the assessment of the long-term outcomes of NAFLD, for instance, cirrhosis, liver cancer, end-stage liver disease and mortality, are still lacking. This investigation may provide more evidence for a risk stratification of NAFLD patients and the early identification of and interventions in NAFLD patients with poor prognosis.

In this study, we examined the association between dietary patterns and ESLD and mortality in NAFLD patients, considering (i) an a priori dietary pattern based on recent dietary priorities for cardiometabolic health [19] and (ii) an a posteriori dietary pattern created by principal component analysis. The combination of a priori and a posteriori patterns may provide a more complete picture for the relation of diet with long-term outcomes of NAFLD.

## 2. Methods

### 2.1. Study Population

The UK Biobank recruited over 502,386 participants aged 37 to 73 years from 22 assessment centers throughout the UK between 2007 and 2010. At baseline, the participants were required to complete a touchscreen questionnaire and a verbal interview, undergo physical measurements and provide biological samples. The UK Biobank received ethics approval from the North West Multicenter Research Ethics Committee (reference no. 16/NW/0274). All participants provided written informed consent at recruitment. This research was conducted using the UK Biobank resource under application number 79302.

Participants with NAFLD at baseline were identified by the fatty liver index (FLI), which has an accuracy of 0.84 in detecting fatty liver, and an FLI > 60 indicates the presence of fatty liver. We then excluded patients with excessive alcohol drinking (alcohol consumption ≥ 30 g/day for men and ≥20 g/day for women) and subjects with other liver diseases (viral hepatitis, Wilson’s disease, hemochromatosis and autoimmune hepatitis). After further exclusion of those with ESLD and missing values of covariates at baseline, 128,695 participants with NAFLD were included in the final analysis (Supplementary Figure S1).

### 2.2. Assessment of Dietary Quality

At the recruitment assessment-center visit, each participant was asked to complete a brief touchscreen food frequency questionnaire (FFQ) with 47 dietary items covering the types and the frequency of consumption of food groups and drinks over the past year. Then, we created a diet quality score based on 10 foods [19]: vegetables, fruits, fish, dairy, whole grains, vegetable oils, refined grains, processed meats, unprocessed red meats and sugar-sweetened beverages, which was used to assess the adherence to ideal dietary patterns in patients with cardiometabolic disease (Supplementary Table S1). Each dietary component was scored from 0 (unhealthiest) to 10 (healthiest) points, and the total diet quality score was the sum of all the diet component scores and ranged from 0 to 100, with a higher score representing a higher overall diet quality. 

### 2.3. Assessment of Dietary Patterns

To derive dietary patterns, the SAS “proc factor” command was used for principal component analysis (PCA) with varimax rotation. When determining the number of principal components to retain, three selection criteria were used: (i) eigenvalue greater than 1, (ii) the scree plot (Supplementary Figure S2) and (iii) the interpretable variance percentage (Supplementary Table S2). Then, the principal components were named based on the food groups that had rotated factor loadings with an absolute value ≥ 0.3. Finally, three dietary patterns were identified for analysis: (i) meat pattern (abundant in red meat and poultry), (ii) prudent (abundant in fruit, vegetable and fish) and (iii) high-quality carbohydrate (high in whole grains, but low in refined grains).

### 2.4. Ascertainment of Outcomes

The date of death was obtained from death certificates held by the National Health Service (NHS) Information Centre (England and Wales) and the NHS Central Register Scotland (Scotland). Dates and causes of hospital admission were identified via record linkage to Health Episode Statistics (England and Wales) and the Scottish Morbidity Records (Scotland). Prevalent and incident ESLD cases within the UK Biobank were ascertained through data linkage to hospital inpatient admissions and death registries. We defined incident ESLD according to the ICD-10 (international classification of diseases, 10th revision) codes K74.6, K76.6, K76.7, I85.0, I85.9, I86.4, I98.2, I98.3, R18, Z94.4, C22.0. Another outcome of the current study included all-cause and three types of cause-specific mortality [liver-related (ICD-10 codes K70-K76), cardiovascular disease (CVD)-related (ICD-10 codes I20, I21, I25, I48, I50, I60, I61, I63, and I64) and cancer-related (ICD-10 codes C00-C97), Supplementary Table S3).

At the time of the analysis, the updating dates of linkages to hospital inpatient admissions and death registries were 30 September 2021 and 31 October 2021, respectively. The follow-up time in person-y was calculated from the date of attendance until the date of ESLD diagnosis, loss to follow-up or death, whichever occurred earlier.

### 2.5. Covariates

Information on demographic factors and lifestyle factors were collected using a touch-screen, self-completed questionnaire at the baseline assessment visit for the UK Biobank. The Townsend deprivation index was used as a measure of the socioeconomic status and to categorize the sample population into quintiles from the least deprived (quintile 1 to the most deprived (quintile 5). To measure the total sedentary time, the sum of self-reported hours spent watching television and using the computer was derived on a typical day. Sedentary behavior was defined as sedentary time > 4 h. The body mass index (BMI) value was obtained from the weight divided by the square of the height in meters. Hypertension was defined as systolic pressure ≥ 140 mmHg, diastolic pressure ≥ 90 mmHg, use of medications for blood pressure or as self-reported or diagnosed by a doctor. Diabetes was defined as blood glucose ≥ 11.1 mmol/L, glycated hemoglobin (HbA1c) ≥ 48 mmol/mol, use of insulin or as self-reported or diagnosed by a doctor. Alanine aminotransferase, triglycerides and cholesterol were measured in blood samples collected at recruitment on a Beckman Coulter AU5800. The UK Biobank performed detailed quality control and correction for technical outliers. 

### 2.6. Statistical Analysis

Baseline sociodemographic, lifestyle and other characteristics were summarized across diet score quartiles. The categorical variables are displayed as percentages and were tested by chi-squared tests. Continuous variables are displayed as means with standard deviations (SDs) and were tested by one-way ANOVA. The associations of diet quality and derived patterns with incident ESLD, all-cause mortality and cause-specific mortality were investigated using Cox-proportional hazard models. Hazard ratios (HR) and 95% confidence intervals (CI) for each quartile of exposure were calculated. Model 1 was adjusted for age, sex, ethnicity, Townsend deprivation index (quintiles), education level (university/college degree or others), household income (less than £18,000, £18,000 to £30,999, £31,000 to £51,999, £52,000 to £100,000, greater than £100,000 or do not know/prefer not to answer), and model 2 was adjusted for model 1 plus self-reported smoking status (never, former or current smoker), sedentary behavior, body mass index, baseline diabetes, baseline hypertension, serum alanine aminotransferase, triglycerides and cholesterol. Then, we used multivariate cubic regression splines with 3 knots (10th, 50th, 90th) to visualize the potential nonlinear associations of dietary patterns with incident ESLD and all-cause mortality discovered in the Cox model above by SAS macro *%RCS_Reg*. To examine the overall statistical significance as well as the non-linearity of the exposures, we used likelihood ratio tests. We then investigated whether these associations differed by age, sex and other factors by performing a subgroup analysis and fitting an interaction term to the model. The hazard ratio of the product term was the measure of the interaction on the multiplicative scale. Sensitivity analyses were performed by excluding individuals with incident ESLD or who died within 2 years, those who had extreme BMIs (BMI < 15 or >40 kg/m^2^), those who made any major changes to their diet in the last 5 years and those whose diet varied much from week to week. SAS 9.4 was used for all analyses. All statistical tests were 2-sided, and *p* < 0.05 was defined as statistically significant. 

## 3. Results

### 3.1. Baseline Characteristics

The baseline characteristics of participants by diet quality score quintiles are shown in Table 1. At baseline, the participants with a higher diet quality tended to be female, older, of White ethnicity, less socially deprived and more educated. In addition, they were less often current smokers and spent less time sitting still. They also had lower levels of alanine aminotransferase, gamma glutamyl transferase, triglycerides and total cholesterol. Interestingly, those NAFLD patients with a higher diet quality were more likely to suffer from comorbid hypertension and type 2 diabetes.

### 3.2. Association of Diet Quality with Incident ESLD and Mortality

During a median of 12.5 years follow-up (1,569,342 person-years), 1925 ESLD and 12,466 deaths occurred. In Table 2, compared with the patients in the lowest quintile, those in the highest quintile of the diet quality had 16% lower odds of ESLD and 18% lower odds of all-cause mortality after adjustments for covariates in model 1. After further adjusting for lifestyle and biochemistry factors in model 2, the inverse association remained significant. The HRs (95% CIs) in quintiles 2–5 were 0.95 (0.83–1.08), 0.83 (0.72–0.95), 0.84 (0.73–0.96) and 0.76 (0.66–0.87) for ESLD, and 0.94 (0.89–0.99), 0.91 (0.86–0.96), 0.85 (0.80–0.90) and 0.84 (0.79–0.88) for all-cause mortality, respectively. The associations of diet quality with cause-specific mortality were concluded in Supplementary Table S4. Similarly, a higher diet quality significantly reduced the risk of liver, CVD and cancer mortality [HR_Q5vsQ1_: 0.53 (0.33–0.85), 0.88 (0.78–0.98) and 0.85 (0.79–0.93), respectively].

### 3.3. Association of Dietary Patterns with Incident ESLD and Mortality

**Figure 1:**
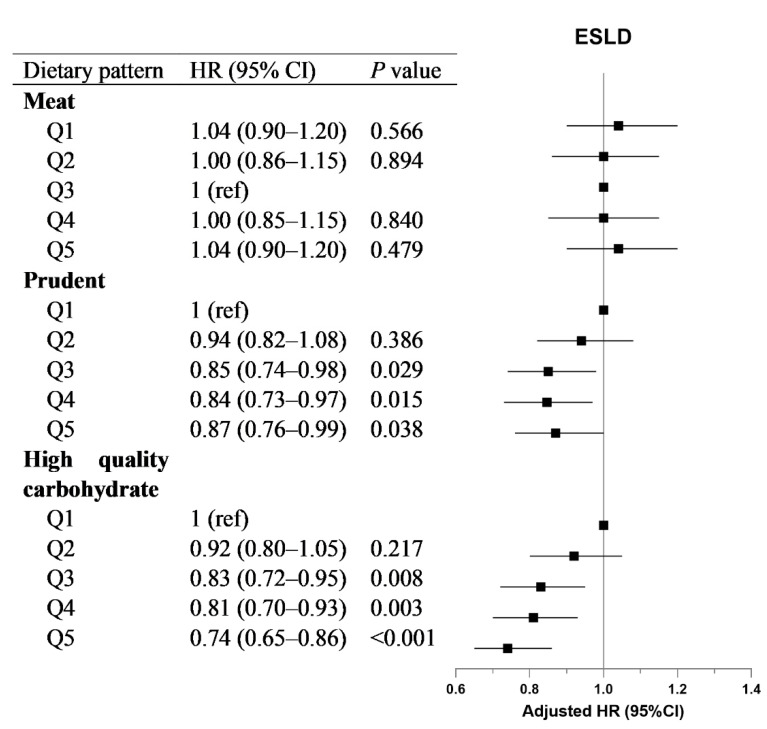
**Association of dietary patterns with incident ESLD.** For the meat diet pattern, the quintile with the lowest hazard ratio (Q3) was set as a reference. The model was adjusted for age, sex, ethnicity, Townsend deprivation index (quintiles), education level (university/college degree or others), household income (less than £18,000, £18,000 to £30,999, £31,000 to £51,999, £52,000 to £100,000, greater than £100,000 or do not know/prefer not to answer), self-reported smoking status (never, former or current smoker), sedentary behavior, body mass index, baseline diabetes, baseline hypertension, serum alanine aminotransferase, triglycerides and cholesterol.

The associations between dietary patterns and ESLD risk are shown in Figure 1. We observed that a high-quality carbohydrate dietary pattern was negatively correlated with the risk of ESLD [HR_Q5vsQ1_: 0.74 (0.65–0.86)]. In addition, a prudent dietary pattern showed a non-linear negative association with the risk of ESLD; the HRs (95% CIs) in quintiles 2–5 were 0.94 (0.82–1.08), 0.85 (0.74–0.98), 0.84 (0.73–0.97), and 0.87 (0.76–0.99). However, we did not find a significant association with the meat-rich dietary pattern.

**Figure 2:**
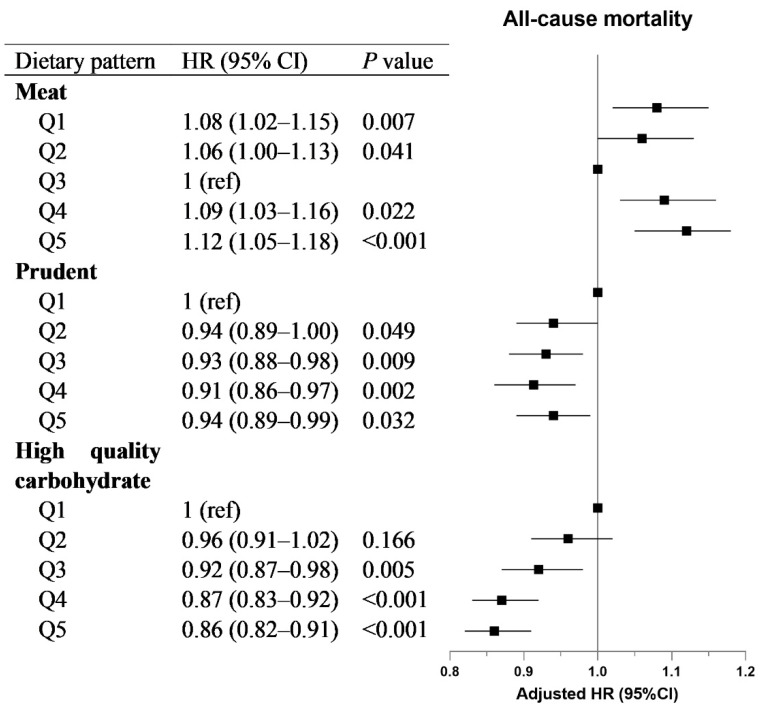
**Association of dietary patterns with all-cause mortality.** For the meat diet pattern, the quintile with the lowest hazard ratio (Q3) was set as a reference. The model was adjusted for age, sex, ethnicity, Townsend deprivation index (quintiles), education level (university/college degree or others), household income (less than £18,000, £18,000 to £30,999, £31,000 to £51,999, £52,000 to £100,000, greater than £100,000, or do not know/prefer not to answer), self-reported smoking status (never, former or current smoker), sedentary behavior, body mass index, baseline diabetes, baseline hypertension, serum alanine aminotransferase, triglycerides and cholesterol.

The results of the relation between the dietary patterns and mortality are shown in Figure 2. Here, we revealed a U-shaped association of the meat-rich dietary pattern with all-cause mortality. Compared with quintile 3, the HRs (95% CIs) in quintiles 1–2 were 1.08 (1.02–1.15) and 1.06 (1.00–1.13); the HRs (95% CIs) in quintiles 4–5 were 1.09 (1.03–1.16) and 1.12 (1.05–1.18). Similar to what observed for ESLD, a prudent dietary pattern and high-quality carbohydrate dietary pattern also demonstrated negative associations with all-cause mortality. The associations of these dietary patterns with cause-specific mortality are reported in Supplementary Table S5.

**Figure 3:**
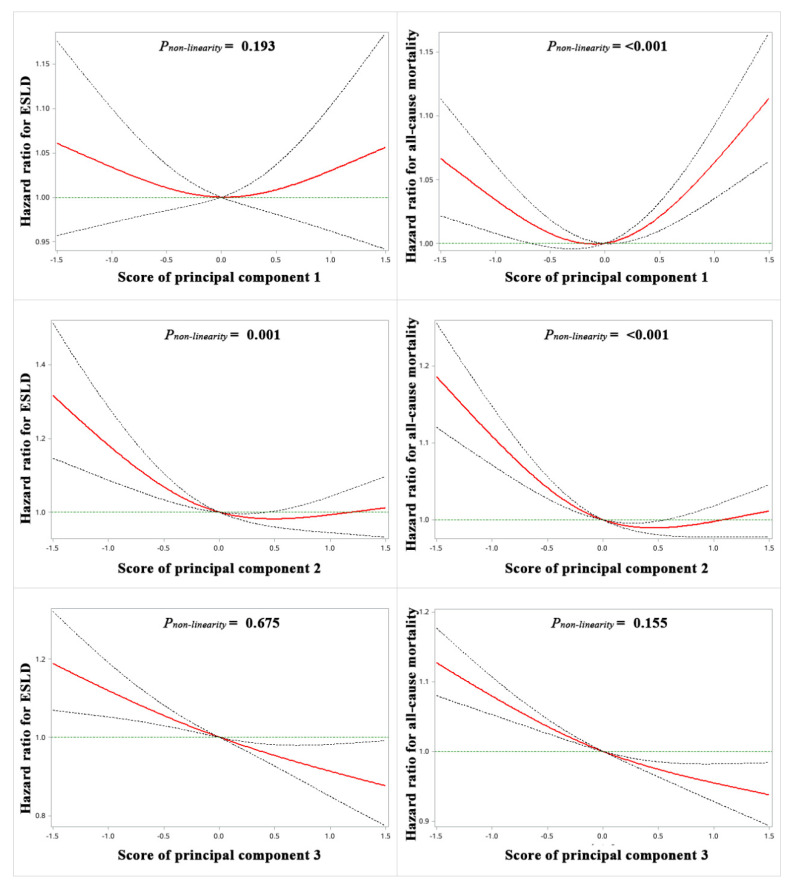
Restricted cubic spline analyses of the relationship between dietary patterns and the incidence of end-stage liver disease (ESLD) and all-cause mortality. Red lines represent adjusted hazard ratios [with 95% CI (dashed lines)] based on restricted cubic splines with knots at the 10th, 50th, 90th. The model was fully adjusted. Principal component 1: meat; principal component 2: prudent; principal component 3: high-quality carbohydrate.

The analysis of cubic splines (Figure 3) also showed the U-shaped association of the meat-rich dietary pattern with all-cause mortality (*p_non-linearity_* < 0.001) and the L-shaped association between a prudent dietary pattern and ESLD as well as all-cause mortality (All *p_non-linearity_* ≤ 0.001). For the high-quality carbohydrate dietary pattern, there was a linear association with ESLD (*p_non-linearity_* = 0.675) and all-cause mortality (*p_non-linearity_* = 0.155).

### 3.4. Subgroup Analyses and Sensitivity Analyses

The subgroup analyses for diet quality according to different risk factors are shown in Supplementary Tables S6 and S7. There were no significant differences across all investigated subgroups for ESLD. For all-cause mortality, we found that a higher diet quality was associated with a decreased risk among current/previous smokers (*p*_interaction_ < 0.002).

We performed a number of sensitivity analyses to examine the robustness of the findings. When we excluded the first 2 years of follow-up, the patients with extreme BMIs, those who made any major changes to their diet in the last 5 years, and those whose diet varied much from week to week, we found that the observed associations of diet quality with ESLD and all-cause mortality remained unchanged (Supplementary Table S8). 

## 4. Discussion

In this large longitudinal study, we observed that dietary patterns were significantly associated with the long-term outcomes of NAFLD. First, a higher a priori-derived diet quality score was inversely related to the risks of ESLD as well as all-cause mortality and liver-, CVD- and cancer-related mortality. Second, a greater adherence to a posteriori-derived dietary patterns (prudent and high-quality carbohydrate patterns) was associated with a lower risk of ESLD in NAFLD patients, whereas this association was nonsignificant for the meat-rich pattern. Third, there was a U-shaped association between the meat-rich pattern and all-cause mortality for NAFLD patients, while this association was negative for the prudent and high-quality carbohydrate patterns.

A large body of cross-sectional studies have reported an inverse relationship of the diet quality score, as assessed by AHEI [20] and MDS [21], with prevalent NAFLD. In addition, whether different dietary patterns were related to NAFLD risks was also investigated, but contradictory results were reached [17]. A cross-sectional study conducted in 229 Brazilian adults demonstrated that a prudent pattern was negatively associated with NAFLD diagnosed by ultrasonography [22]. In contrast, another cross-sectional study covering 999 Chinese patients found this association to be nonsignificant [23]. An Australian prospective cohort study including 995 adolescents observed that a Western dietary pattern high in red and processed meat, soft drinks, refined grains and sauces at age 14 was associated with a 1.59-fold risk of NAFLD three years later [24]. However, till now, these studies were mainly small cross-sectional studies and centered on the prevalent risks of NAFLD. The evidence for a relationship between dietary pattern and long-term outcomes of NAFLD remains sparce. 

In this study, we used a diet quality score which was adopted in previous epidemiological studies of cardiovascular disease [25] and type 2 diabetes [26], created on the basis of recent dietary priorities of cardiometabolic health [19]. In addition, in recent years, the role of dietary patterns generated by principal component analysis has been extensively investigated in observational studies of cardiometabolic disease [27]. They are more representative of dietary patterns in a given population [28]. Therefore, we combined these two methods in this study to provide a more complete picture for the relation of diet with the long-term outcomes of NAFLD. Our analysis showed that NAFLD patients with a higher diet quality carried lowers odds of ESLD and all-cause and cause-specific mortality. In addition, a prudent dietary pattern high in vegetables, fruits and fish was negatively associated with a poor prognosis of NAFLD. This association was also observed for the high-quality carbohydrate dietary pattern which was high in whole grains and low in refined grains. 

For the clinical implications of this study, it provides a more comprehensive understanding of the effects of dietary patterns on the development of severe outcomes of NAFLD. The advanced liver disease in the late course of NAFLD is associated with a severely impaired quality of life and poor prognosis [29]. Given the sheer number of NAFLD patients and the fact that advanced liver disease is usually diagnosed late, a better risk stratification of NAFLD is urgently needed [30]. The early recognition of NAFLD patients with adverse outcomes would allow policy makers and clinicians to plan and implement a more effective secondary prevention [9]. This study showed that NAFLD patients may benefit from a high diet quality and prudent and high-quality carbohydrate dietary patterns. Conversely, NAFLD patients with other diet patterns may be more likely to suffer from adverse health outcomes and warrant more close attention during regular follow-up.

There are several possible mechanisms linking the dietary patterns with the long-term outcomes of NAFLD. The prudent dietary pattern has been shown to have beneficial effects on NAFLD due to its anti-inflammatory, anti-fibrosis and antioxidant capacity [31]. Carotenoids and polyphenols are two major antioxidants that are abundant in vegetables and fruits. In experimental studies of NAFLD models, they improved insulin sensitivity, accelerated *β*-oxidation and repressed de novo lipogenesis [32]. Furthermore, they inhibited the activation of hepatic stellate cells and therefore ameliorated carcinogenesis [32]. Omega-3 poly-unsaturated fatty acids contained in fish oil can alleviate insulin resistance, reduce hepatic lipid accumulation and improve steatohepatitis [33,34]. The mechanism through which whole grains exert favorable impacts on NAFLD is multifaced. First, wheat bran, a more abundant compound in whole grains than in refined grains, reduced the liver triglyceride content in an in vivo model of metabolic syndrome [35]. Second, several phytochemicals that are significantly reduced after grain refining can promote the synthesis of VLDL and thus export lipids outside the liver [36]. Third, whole grains may display beneficial effects on the composition of the gut microbiota [37], which may influence the progression of NAFLD through the gut–liver axis [38]. 

This study has several limitations that warrant discussion. First, we selected several important diet constituents to create a dietary quality score based on the recent guidelines of cardiometabolic health. However, other components may also display a key role in the progression of NAFLD. Second, we analyzed the association between dietary patterns at baseline and the risk of adverse outcomes of NAFLD. The dietary patterns were not assessed during the follow-up. We were unable to assess the longitudinal dynamic change in dietary patterns, which may more closely reflect the habitual eating in real-world life. Third, as with all observational studies, we were unable to draw causality about the relationship between dietary patterns and long-term outcomes of NAFLD. The only way to clearly measure this relationship is through experimental designs. 

## 5. Conclusions

In conclusion, higher diet quality and greater adherence to a prudent dietary pattern rich in vegetables, fruits and fish were associated with a lower likelihood of ESLD and mortality in NAFLD patients. High-quality carbohydrate dietary patterns showed the same association. NAFLD patients with inappropriate meat dietary patterns had a higher risk of adverse outcomes. These findings need to be confirmed with further interventional studies to assess whether the improvement of the dietary patterns is effective in the primary and secondary prevention of NAFLD.

## Figures and Tables

**Table 1:** UK Biobank participants’ characteristics by the diet quality score.

Variables	Q1	Q2	Q3	Q4	Q5	*p* Value
Male (%)	70.9	62.4	56.5	53.2	53.7	<0.001
Age (years)	55.7 ± 8.3	56.9 ± 8.1	57.3 ± 7.9	57.9 ± 7.6	59.1 ± 7.1	<0.001
White ethnicity (%)	93.4	91.8	92.1	93.7	94.5	<0.001
Townsend deprivation index	−0.4 ± 3.4	−0.8 ± 3.3	−1.1 ± 3.2	−1.3 ± 3.1	−1.2 ± 3.1	<0.001
College or university degree (%)	17.53	23.13	26.33	30.08	29.79	<0.001
Household income (£)						<0.001
<18,000	26.5	25.0	22.8	22.5	24.8	
18,000 to 30,999	22.2	22.1	22.5	22.5	23.6	
31,000 to 51,999	20.3	20.5	20.7	21.1	20.5	
52,000 to 100,000	12.5	13.7	15.0	15.4	13.5	
>100,000	2.2	2.6	3.1	3.6	2.8	
Sedentary behavior (%)	46.5	42.9	40.9	39.1	39.1	<0.001
Smoking status (%)						<0.001
Never	48.3	52.9	54.7	55.5	53.0	
Previous	34.0	35.7	36.6	37.1	41.0	
Current	17.7	11.4	8.7	7.3	6.0	
Alcohol						
Never or in special occasions only	29.8	29.5	28.5	26.9	28.2	
1 to 3 times/month	15.9	15.2	15.5	15.4	15.0	
1 to 4 times/week	47.7	48.6	49.4	50.6	50.3	
Daily or almost daily	6.6	6.8	6.6	7.0	6.6	
Body mass index (kg/m^2^)	31.6 ± 4.7	31.8 ± 4.6	31.9 ± 4.6	31.8 ± 4.6	31.9 ± 4.6	<0.001
Waist circumference (cm)	103.5 ± 10.2	102.9 ± 10.2	102.5 ± 10.1	102.2 ± 10.1	102.3 ± 10.1	<0.001
Hypertension (%)	68.2	69.4	69.4	70.0	72.9	<0.001
Diabetes (%)	10.0	11.2	12.2	12.2	17.4	<0.001
Alanine aminotransferase (U/L)	30.3 ± 17.0	29.5 ± 16.5	29.1 ± 17.4	28.9 ± 16.1	28.8 ± 16.6	<0.001
Gamma glutamyltransferase (U/L)	52.3 ± 52.3	50.6 ± 48.8	49.3 ± 49.8	48.0 ± 45.9	47.8 ± 48.4	<0.001
Triglycerides (mmol/L)	2.5 ± 1.2	2.4 ± 1.2	2.4 ± 1.2	2.3 ± 1.1	2.3 ± 1.1	<0.001
Total cholesterol (mmol/L)	5.7 ± 1.2	5.7 ± 1.2	5.7 ± 1.2	5.7 ± 1.2	5.6 ± 1.3	<0.001

Quintiles of the diet score: Q1, ≤40.54; Q2, 40.55–48.66; Q3, 48.67–56.11; Q4, 56.12–63.61; and Q5, ≥63.62. Values are the mean (±standard deviation, SD) or percentage (%) and were examined by one-way ANOVA or chi-square test. Post hoc analysis (Bonferroni method) showed significant differences for Q2–Q5 compared with Q1, except for total cholesterol.

**Table 2:** HRs of ESLD and all-cause mortality for quintiles of the diet quality score.

Diet Score	ESLD			All-Cause Mortality		
	HR (95% CI)	*p* Value	*p*_trend_	HR (95% CI)	*p* Value	*p*_trend_
Model 1			0.001			0.004
Q1	1 (ref)			1 (ref)		
Q2	0.95 (0.83–1.09)	0.497		0.92 (0.87–0.97)	0.003	
Q3	0.86 (0.75–0.99)	0.035		0.88 (0.84–0.94)	<0.001	
Q4	0.85 (0.74–0.98)	0.027		0.84 (0.80–0.89)	<0.001	
Q5	0.84 (0.73–0.96)	0.010		0.82 (0.77–0.87)	<0.001	
Model 2			<0.001			<0.001
Q1	1 (ref)			1 (ref)		
Q2	0.95 (0.83–1.08)	0.412		0.94 (0.89–0.99)	0.021	
Q3	0.83 (0.72–0.95)	0.008		0.91 (0.86–0.96)	0.001	
Q4	0.84 (0.73–0.96)	0.013		0.85 (0.80–0.90)	<0.001	
Q5	0.76 (0.66–0.87)	<0.001		0.84 (0.79–0.88)	<0.001	

Quintiles of the diet score: Q1, ≤40.54; Q2, 40.55–48.66; Q3, 48.67–56.11; Q4, 56.12–63.61; and Q5, ≥63.62. Model 1 was adjusted for age, sex, ethnicity, Townsend deprivation index (quintiles), education level (university/college degree or others) and household income (less than £18,000, £18,000 to £30,999, £31,000 to £51,999, £52,000 to £100,000, greater than £100,000 or do not know/prefer not to answer). Model 2 was adjusted for model 1 plus self-reported smoking status (never, former or current smoker), sedentary behavior, body mass index, baseline diabetes, baseline hypertension, serum alanine aminotransferase, triglycerides and cholesterol.

## Data Availability

Data described in the manuscript, code book, and analytic code will be made available upon request pending application. This research was conducted using the UK Biobank resource under application number 79302.

## Supplementary Materials

The following supporting information can be downloaded at: https://www.mdpi.com/article/10.3390/nu15020271/s1, Figure S1. Summary of study design and analytical strategy; Figure S2. Scree plot of the factor analysis; Table S1. Components and scaling methods of diet quality score used in the UK Biobank study; Table S2. PCA -derived dietary patterns and their factor loadings; Table S3. Criteria for the ESLD, CVD, Cancer; Table S4. HRs of cause-specific mortality for quintiles of diet quality score; Table S5. HRs of cause-specific mortality for quintiles of dietary patterns; Table S6. Subgroup analyses in diet quality and ESLD; Table S7. Subgroup analyses in diet quality and all-cause mortality; Table S8. Sensitivity analyses of the HRs for the associations of diet quality with ESLD and all-cause mortality.
