# Supplementary material for: Dietary Patterns and Long-Term Outcomes in Patients with NAFLD: A Prospective Analysis of 128,695 UK Biobank Participants
Source: Nutrients. 2023 Jan 5;15(2):271. doi: 10.3390/nu15020271 (PMC9862257; doi:10.3390/nu15020271)
Supplement: Supplementary file 1 [file nutrients-15-00271-s001.zip › nutrients-2122931-supplementary.pdf]

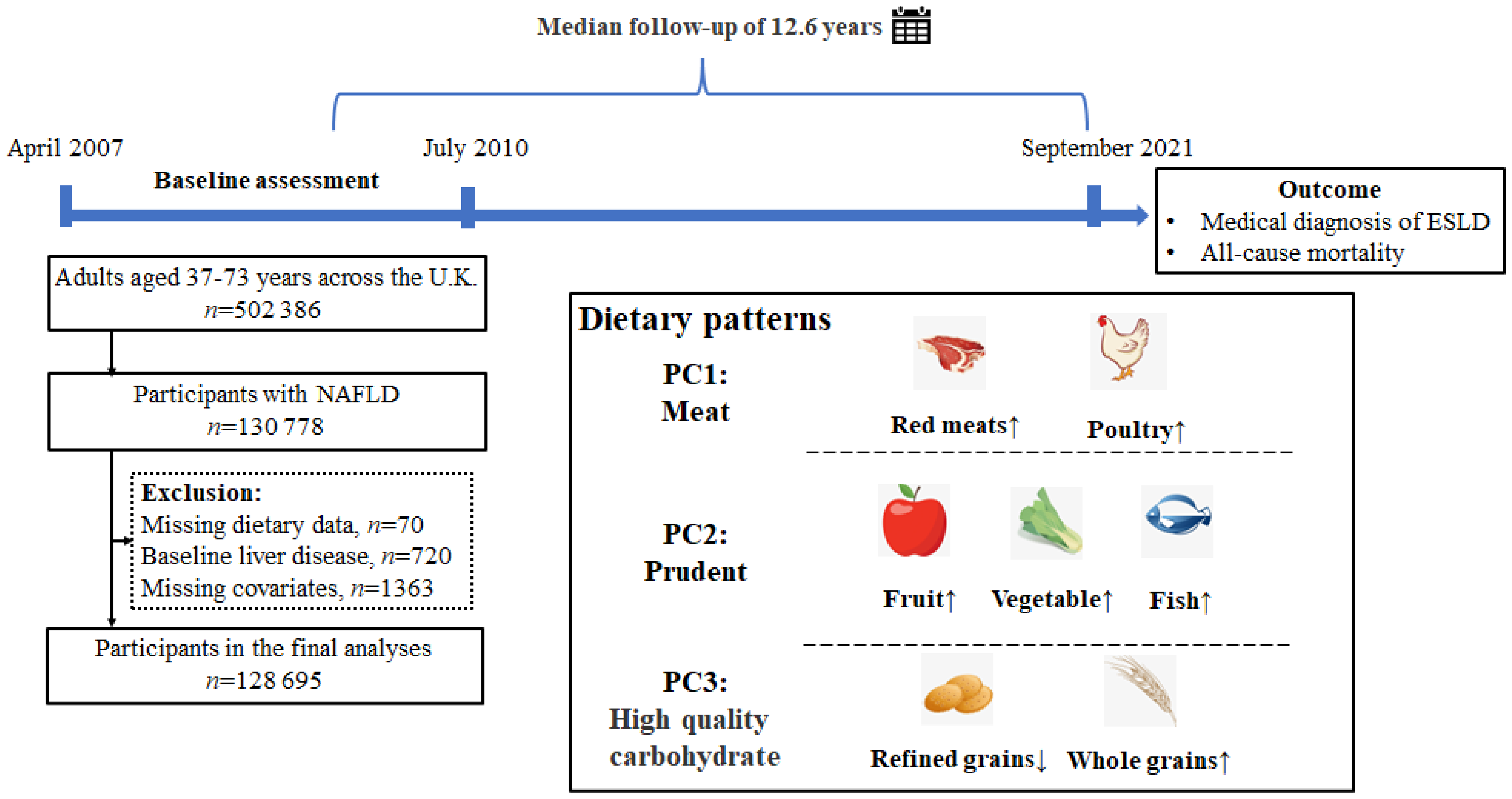

Supplementary Figure S1. Summary of study design and analytical strategy.

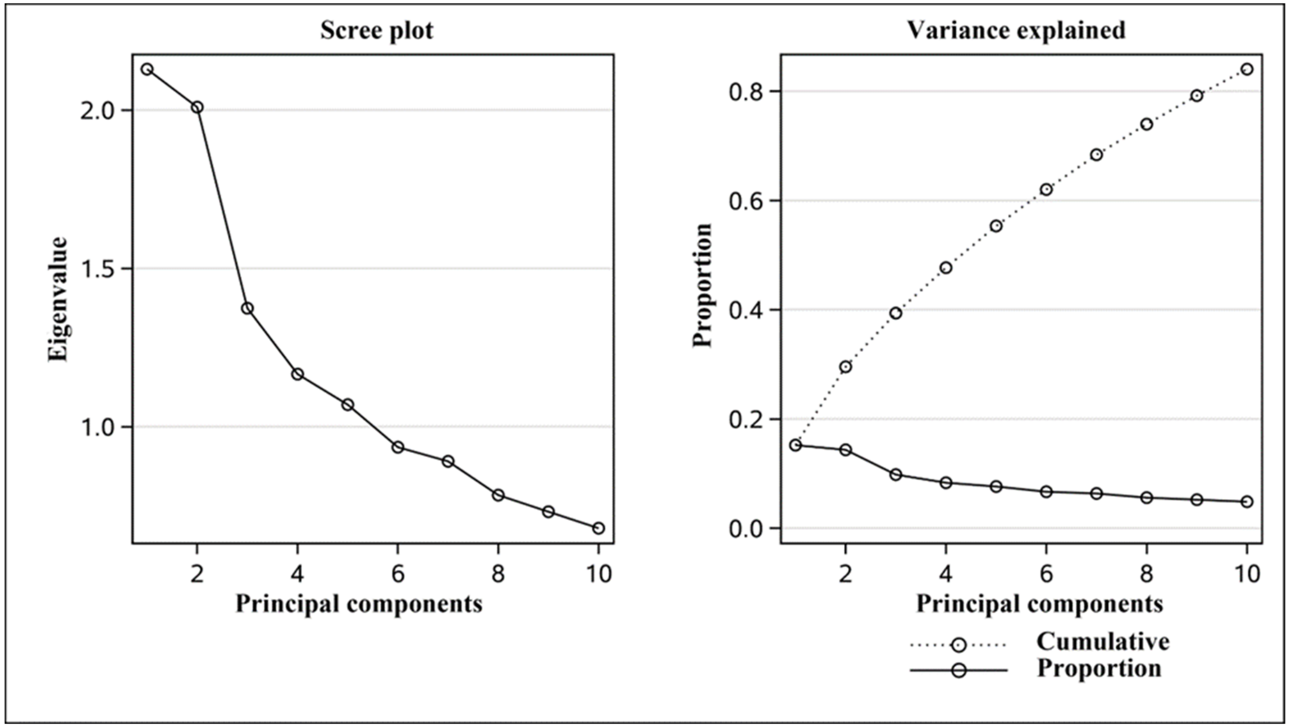

Supplementary Figure S2. Scree plot of the factor analysis.

Supplementary Table S1. Components and scaling methods of diet quality score used in the UK Biobank study

**Components**	**Field IDs**	**Amount per serving**	**Criteria for maximum score (10)**	**Criteria for minimum score (0)**
Fruit	1309 (pieces fresh fruit/day)<br>1319 (pieces dried fruit/day)	1309 – 1 piece<br>1319 – 5 pieces	≥3 servings/day	0 servings/day
Vegetable	1289 (tablespoons cooked vegetables/day)<br>1299 (salad/raw vegetables/day)	3 heaped tablespoons	≥3 servings/day	0 servings/day
Whole grains	1438, 1448 (whole meal/whole grain bread slices/week)<br>1458, 1468 (bran/oat/muesli cereal bowls/week)	1438/1448 – 1 slice/day<br>1458/1468 – 1 bowl/day	≥3 servings/day	0 servings/day
Fish	1329 (oily fish/week)<br>1339 (non-oily fish/week)	Once/week	≥2 servings/week	0 servings/week
Dairy	1408 (cheese/week)<br>1418 (milk type)	1408 – 1 piece/day<br>1418 – 1 glass/day if consumption of anytype of milk	≥2 servings/day	0 servings/day

Vegetable oils	1428 (Flora Pro-Active/Benecol spread)<br>2654 (Flora Pro-Active/Benecol, soft margarine -, olive oil based -, polyunsaturated/sunflower oil based -, other low/reduced fat spread)<br>1438 (bread slices/week)	1 serving/day if in combination with eating at least 2 slices of bread (ID 1438)	≥2 servings/day	0 servings/day
Refined grains	1438<br>1448 (white, brown, other bread slices/week)<br>1458<br>1468 (biscuit, other cereals/week)	1438/1448 – 1 slice/day 1458/1468 – 1 bowl/day	0 servings/day	>2 servings/day
Processed meats	1349 (processed meat/week or daily)<br>3680(age when last ate meat)	1349 – 1 piece/day<br>3680 – 0 pieces/day if indicated having never eaten meat	0 serving/week	>1 serving/week
Unprocessed red meats	1369 (beef/week or day)<br>1379 (lamb or mutton/week or day)<br>1389(pork/week or day)<br>3680 (age when last ate meat)	1359-1389 – once/week<br>3680 – 0 pieces/day if indicated having never eaten meat	0 serving/week	>2 serving/week
Sugar-sweetened beverages	6144 (never consumes drinks containingsugar)	0 servings	Don't drink	Drink

Zhuang, P., Liu, X., Li, Y., Wan, X., Wu, Y., Wu, F., … Jiao, J.. (2021). Effect of Diet Quality and Genetic Predisposition on Hemoglobin A1c and Type 2 Diabetes Risk: Gene-Diet Interaction Analysis of 357,419 Individuals.doi:10.2337/figshare.15085239.v1

Supplementary Table S2. PCA -derived dietary patterns and their factor loadings

Food items	Dietary patterns		
	Meat	Prudent	High quality carbohydrate
Cooked vegetable	0.01949	0.65704	-0.10995
Salad / raw vegetable	-0.06781	0.69771	-0.10208
Fresh fruit	-0.09319	0.57226	0.05454
Dried fruit	-0.08238	0.39036	0.11591
Oily fish	0.19224	0.49596	0.24435
Non-oily fish	0.28878	0.39028	0.16236
Processed meat	0.55157	-0.22960	-0.11822
Poultry	0.49302	0.13188	0.05392
Beef	0.67182	-0.06005	-0.04359
Lamb/mutton	0.63600	0.08068	-0.01525
Pork	0.67022	-0.01353	-0.00354
Cheese	0.13109	-0.07946	0.11892
Refined grains*	0.10574	-0.13497	-0.83423
Whole grains*	-0.02567	0.07911	0.85407
Explained variance, %	15.21	29.56	39.38

Food items were standardized and included in the principal component analysis (PCA) with varimax rotation. Rotated factor loadings with an absolute value ≥|0.30| were considered to significantly contribute to the dietary pattern and colored red.

* The definitions of refined grains and whole grains were based on the criteria in Supplementary Table S1.

Supplementary Table S3. Criteria for the ESLD, CVD, Cancer

End stage liver disease		CVD death		Liver death	
**Code**	**Description**	**Code**	**Description**	**Code**	**Description**
K74.6	Other and unspecified cirrhosis of liver	I20	Angina pectoris	K70	Alcoholic liver disease
K76.6	Portal hypertension	I21	Acute myocardial infarction	K71	Toxic liver disease
K76.7	Hepatorenal syndrome	I25	Chronic ischaemic heart disease	K72	Hepatic failure, not elsewhere classified
I85.0	Oesophageal varices with bleeding	I48	Atrial fibrillation and flutter	K74	Fibrosis and cirrhosis of liver
I85.9	Oesophageal varices without bleeding	I50	Heart failure	K75	Other inflammatory liver diseases
I86.4	Gastric varices	I60	Subarachnoid haemorrhage	K76	Other diseases of liver
I98.2	Oesophageal varices in diseases classified elsewhere	I61	Intracerebral haemorrhage		
I98.3	Oesophageal varices with bleeding in diseases classified elsewhere	I63	Cerebral infarction	**Cancer death**	
R18	Ascites	I64	Stroke, not specified as haemorrhage or infarction	C00-C97	Malignant neoplasms
Z94.4	Liver transplant				
C22.0	Liver cell carcinoma				

Supplementary Table S4. HRs of cause-specific mortality for quintiles of diet quality score (n=128 549)

Diet quality score	Liver			CVD			Cancer		
	HR (95% CI)	*P* value	P_{trend}	HR (95% CI)	*P* value	P_{trend}	HR (95% CI)	*P* value	P_{trend}
**Model 1**			0.145			0.142			0.005
Q1	1 (ref)			1 (ref)			1 (ref)		
Q2	0.80 (0.51–1.25)	0.324		0.99 (0.88–1.10)	0.811		0.92 (0.85–1.00)	0.045	
Q3	0.78 (0.49–1.23)	0.279		0.97 (0.87–1.09)	0.620		0.95 (0.87–1.03)	0.176	
Q4	0.76 (0.48–1.22)	0.253		0.84 (0.75–0.95)	0.004		0.91 (0.84–0.99)	0.020	
Q5	0.70 (0.44–1.12)	0.132		0.98 (0.87–1.09)	0.670		0.88 (0.81–0.96)	0.003	
**Model2**			0.009			0.001			<0.001
Q1	1 (ref)			1 (ref)			1 (ref)		
Q2	0.75 (0.48–1.17)	0.206		0.96 (0.86–1.07)	0.483		0.91 (0.84–0.99)	0.029	
Q3	0.67 (0.42–1.06)	0.086		0.93 (0.83–1.04)	0.201		0.94 (0.86–1.01)	0.105	
Q4	0.66 (0.42–1.06)	0.085		0.80 (0.71–0.90)	<0.001		0.89 (0.82–0.97)	0.008	
Q5	0.53 (0.33–0.85)	0.009		0.88 (0.78–0.98)	0.021		0.85 (0.79–0.93)	<0.001	

Model 1 was adjusted for age, sex, ethnicity, Townsend deprivation index (quintiles), education level (university/college degree or others), and household income (less than £18,000, £18,000 to £30,999, £31,000 to £51,999, £52,000 to £100,000, greater than £100,000, or do not know/prefer not to answer).

Model 2 was adjusted for model 1 plus self-reported smoking status (never, former or current smoker), sedentary behavior, body mass index, baseline diabetes, baseline hypertension, serum alanine aminotransferase, triglycerides, and cholesterol.

Supplementary Table S5. HRs of cause-specific mortality for quintiles of dietary patterns

Dietary pattern	Liver			CVD			Cancer		
	HR (95% CI)	*P* value	P_{trend}	HR (95% CI)	*P* value	P_{trend}	HR (95% CI)	*P* value	P_{trend}
**Meat**			0.909			0.861			0.008
Q1	1.25 (0.74–2.09)	0.404		1.18 (1.04–1.33)	0.017		0.98 (0.89–1.07)	0.940	
Q2	1.06 (0.62–1.80)	0.833		1.10 (0.97–1.24)	0.268		1.09 (1.00–1.19)	0.050	
Q3	1 (ref)			1 (ref)			1 (ref)		
Q4	1.24 (0.75–2.06)	0.399		1.12 (0.99–1.26)	0.058		1.07 (0.98–1.16)	0.133	
Q5	1.16 (0.70–1.91)	0.568		1.11 (0.98–1.25)	0.072		1.12 (1.03–1.21)	0.002	
**Prudent**			0.047			0.425			0.077
Q1	1 (ref)			1 (ref)			1 (ref)		
Q2	0.69 (0.44–1.09)	0.116		1.00 (0.89–1.12)	0.960		0.96 (0.88–1.04)	0.286	
Q3	0.55 (0.34–0.90)	0.017		0.95 (0.84–1.07)	0.389		0.97 (0.89–1.05)	0.401	
Q4	0.59 (0.37–0.94)	0.027		0.88 (0.78–0.99)	0.039		0.89 (0.82–0.97)	0.009	
Q5	0.65 (0.41–1.03)	0.065		1.00 (0.89–1.13)	0.969		0.95 (0.87–1.03)	0.208	
**High quality carbohydrate**			0.002			0.001			0.044
Q1	1 (ref)			1 (ref)			1 (ref)		
Q2	0.81 (0.53–1.25)	0.347		0.89 (0.80–0.99)	0.037		1.02 (0.94–1.10)	0.653	
Q3	0.74 (0.47–1.16)	0.189		0.84 (0.75–0.95)	0.004		0.95 (0.87–1.03)	0.226	
Q4	0.58 (0.36–0.95)	0.029		0.78 (0.69–0.87)	<0.001		0.93 (0.85–1.01)	0.086	
Q5	0.50 (0.31–0.81)	0.005		0.84 (0.75–0.93)	0.002		0.95 (0.88–1.03)	0.217	

Model was adjusted for age, sex, ethnicity, Townsend deprivation index (quintiles), education level (university/college degree or others), household income (less than £18,000, £18,000 to £30,999, £31,000 to £51,999, £52,000 to £100,000, greater than £100,000, or do not know/prefer not to answer), self-reported smoking status (never, former or current smoker), sedentary behavior, body mass index, baseline diabetes, baseline hypertension, serum alanine aminotransferase, triglycerides, and cholesterol.

Supplementary Table S6. Subgroup analyses in diet quality and ESLD

Subgroups	Q1	Q2	Q3	Q4	Q5	$P_{interaction}$
**Gender**						0.725
**Male**	1 (ref)	0.90 (0.72–1.12)	0.69 (0.55–0.87)	0.73 (0.58–0.91)	0.78 (0.63–0.97)	
**Female**	1 (ref)	0.97 (0.82–1.15)	0.95 (0.80–1.13)	0.93 (0.78–1.11)	0.74 (0.61–0.89)	
**Age**						0.163
**≤60 years**	1 (ref)	1.04 (0.84–1.29)	0.89 (0.71–1.11)	0.92 (0.73–1.16)	0.87 (0.68–1.10)	
**>60 years**	1 (ref)	0.90 (0.75–1.06)	0.81 (0.68–0.96)	0.80 (0.67–0.96)	0.72 (0.61–0.86)	
**Townsend index**						0.065
**Below median**	1 (ref)	1.03 (0.83–1.28)	0.87 (0.70–1.09)	0.89 (0.71–1.11)	0.88 (0.71–1.10)	
**Above median**	1 (ref)	0.92 (0.77–1.09)	0.84 (0.70–1.01)	0.84 (0.70–1.00)	0.70 (0.58–0.84)	
**Education level**						0.076
**Others**	1 (ref)	0.90 (0.78–1.04)	0.78 (0.67–0.91)	0.81 (0.69–0.94)	0.74 (0.63–0.86)	
**College**	1 (ref)	1.43 (0.97–2.09)	1.32 (0.90–1.93)	1.24 (0.86–1.81)	1.12 (0.77–1.63)	
**Smoking**						0.981
**Current or previous**	1 (ref)	0.95 (0.80–1.13)	0.82 (0.68–0.98)	0.88 (0.73–1.06)	0.76 (0.63–0.91)	
**Never**	1 (ref)	0.95 (0.77–1.17)	0.87 (0.71–1.08)	0.82 (0.66–1.01)	0.79 (0.63–0.97)	
**Sedentary behavior**						0.993
**No**	1 (ref)	0.94 (0.78–1.13)	0.87 (0.72–1.06)	0.90 (0.75–1.10)	0.77 (0.63–0.94)	
**Yes**	1 (ref)	0.97 (0.80–1.18)	0.82 (0.67–1.00)	0.81 (0.66–1.00)	0.79 (0.65–0.96)	
**Obesity**						0.161
**No**	1 (ref)	0.97 (0.74–1.27)	0.97 (0.74–1.28)	0.96 (0.73–1.27)	0.85 (0.64–1.13)	
**Yes**	1 (ref)	0.95 (0.81–1.11)	0.81 (0.69–0.95)	0.82 (0.70–0.96)	0.75 (0.64–0.89)	
**Hypertension**						0.691
**No**	1 (ref)	0.95 (0.71–1.27)	0.76 (0.56–1.04)	0.89 (0.66–1.20)	0.75 (0.55–1.03)	
**Yes**	1 (ref)	0.95 (0.82–1.11)	0.86 (0.74–1.01)	0.84 (0.72–0.98)	0.78 (0.66–0.91)	
**Diabetes**						0.760
**No**	1 (ref)	0.93 (0.80–1.08)	0.83 (0.71–0.97)	0.83 (0.71–0.98)	0.75 (0.64–0.89)	
**Yes**	1 (ref)	1.02 (0.78–1.34)	0.88 (0.66–1.17)	0.90 (0.68–1.19)	0.82 (0.63–1.07)	

Model was fully adjusted

Supplementary Table S7. Subgroup analyses in diet quality and all-cause mortality

Subgroups	Q1	Q2	Q3	Q4	Q5	$P_{interaction}$
**Gender**						0.533
**Male**	1 (ref)	0.98 (0.88–1.08)	0.92 (0.83–1.02)	0.90 (0.81–1.00)	0.90 (0.82–1.00)	
**Female**	1 (ref)	0.93 (0.87–0.99)	0.93 (0.87–0.99)	0.84 (0.78–0.90)	0.84 (0.78–0.90)	
**Age**						0.089
**≤60 years**	1 (ref)	1.00 (0.90–1.11)	0.97 (0.87–1.07)	0.96 (0.86–1.06)	0.92 (0.83–1.03)	
**>60 years**	1 (ref)	0.92 (0.86–0.98)	0.90 (0.84–0.96)	0.82 (0.77–0.88)	0.83 (0.78–0.89)	
**Townsend index**						0.059
**Below median**	1 (ref)	0.96 (0.88–1.05)	0.91 (0.84–1.00)	0.90 (0.82–0.98)	0.88 (0.81–0.96)	
**Above median**	1 (ref)	0.94 (0.88–1.01)	0.94 (0.87–1.01)	0.83 (0.77–0.90)	0.85 (0.79–0.91)	
**Education level**						0.837
**Others**	1 (ref)	0.95 (0.90–1.01)	0.93 (0.88–0.99)	0.87 (0.81–0.92)	0.86 (0.81–0.92)	
**College**	1 (ref)	0.92 (0.79–1.06)	0.88 (0.76–1.02)	0.82 (0.71–0.94)	0.82 (0.72–0.95)	
**Smoking**						<0.001
**Current or previous**	1 (ref)	0.93 (0.87–1.00)	0.89 (0.83–0.95)	0.80 (0.74–0.86)	0.82 (0.76–0.88)	
**Never**	1 (ref)	0.98 (0.89–1.08)	1.00 (0.91–1.10)	0.97 (0.89–1.07)	0.94 (0.86–1.04)	
**Sedentary behavior**						0.735
**No**	1 (ref)	0.95 (0.88–1.03)	0.92 (0.85–0.99)	0.86 (0.79–0.93)	0.87 (0.80–0.94)	
**Yes**	1 (ref)	0.94 (0.87–1.02)	0.93 (0.86–1.01)	0.86 (0.80–0.94)	0.85 (0.79–0.92)	
**Obesity**						0.427
**No**	1 (ref)	0.94 (0.85–1.04)	0.89 (0.80–0.99)	0.83 (0.75–0.93)	0.85 (0.77–0.95)	
**Yes**	1 (ref)	0.95 (0.89–1.02)	0.94 (0.88–1.00)	0.88 (0.82–0.94)	0.87 (0.81–0.93)	
**Hypertension**						0.979
**No**	1 (ref)	0.97 (0.86–1.10)	0.93 (0.82–1.05)	0.82 (0.72–0.94)	0.85 (0.75–0.97)	
**Yes**	1 (ref)	0.94 (0.88–1.00)	0.92 (0.86–0.98)	0.87 (0.81–0.92)	0.86 (0.81–0.91)	
**Diabetes**						0.962
**No**	1 (ref)	0.94 (0.88–0.99)	0.86 (0.81–0.92)	0.84 (0.79–0.90)	0.85 (0.80–0.91)	
**Yes**	1 (ref)	0.99 (0.88–1.11)	1.13 (1.00–1.27)	0.91 (0.81–1.03)	0.90 (0.80–1.01)	

Model was fully adjusted

Supplementary Table S8. Sensitivity analyses of the HRs for the associations of diet quality with ESLD and all-cause mortality

	Exclude the first 2 years of follow-up	Exclude extreme BMIs	Exclude major diet changes	Exclude frequent diet variance
ESLD				
Q1	1 (ref)	1 (ref)	1 (ref)	1 (ref)
Q2	0.94 (0.82–1.08)	0.96 (0.84–1.11)	1.01 (0.83–1.23)	0.97 (0.84–1.11)
Q3	0.82 (0.71–0.95)	0.86 (0.74–1.00)	0.87 (0.70–1.07)	0.80 (0.69–0.93)
Q4	0.83 (0.72–0.96)	0.85 (0.73–0.98)	0.92 (0.75–1.14)	0.86 (0.74–0.99)
Q5	0.74 (0.64–0.85)	0.77 (0.66–0.89)	0.79 (0.64–0.97)	0.79 (0.68–0.91)
All-cause mortality				
Q1	1 (ref)	1 (ref)	1 (ref)	1 (ref)
Q2	0.94 (0.89–0.99)	0.96 (0.91–1.01)	0.94 (0.87–1.02)	0.96 (0.90–1.01)
Q3	0.92 (0.87–0.98)	0.92 (0.87–0.98)	0.84 (0.78–0.92)	0.93 (0.87–0.98)
Q4	0.85 (0.80–0.91)	0.86 (0.81–0.91)	0.85 (0.78–0.92)	0.86 (0.81–0.91)
Q5	0.85 (0.80–0.90)	0.85 (0.80–0.90)	0.83 (0.76–0.90)	0.84 (0.80–0.90)

Model was fully adjusted
